# Conventional one-handed compared to two-handed endoscopic ear surgery using an endoscope holder: a single center study

**DOI:** 10.1007/s00405-024-09018-9

**Published:** 2024-10-15

**Authors:** Christoph Müller, Anastasia Raczynski, Susen Lailach, Thomas Zahnert

**Affiliations:** TU Dresden, Faculty of Medicine and University Hospital Carl Gustav Carus, Department of Otorhinolaryngology Head and Neck Surgery, Ear Research Center Dresden, Fetscherstraße 74, Dresden, 01307 Germany

**Keywords:** Two-handed endoscopic ear surgery, Surgical assistance system, Passive endoscope holder, Postoperative outcome, Cut-suture time

## Abstract

**Introduction:**

One-handedness is a challenge in conventional endoscopic ear surgery (EES). We present results on the first-ever application of the passive endoscope holder ‘Endofix exo’ (Co. AKTORmed GmbH, Neutraubling, Germany) in EES, which enables two-handed surgery.

**Methods:**

This two-sided study compares cut-suture time, operating time, postoperative complications, graft take rates, hearing results and quality of life in patients who underwent first stage tympanoplasty due to tympanic membrane perforation with intact ossicular chain conditions. 25 patients received classic EES (EES-, mean age: 28 ± 21 years) and 15 received EES with the passive holder (EES+, mean age: 48 ± 21 years).

**Results:**

Mean operating times (EES-: 96 ± 38 (SD) min; EES+: 107 ± 33 min), cut-suture times (EES-: 68 ± 30 min; EES+: 73 ± 31 min), complications, graft take rates and hearing results (preoperative air bone gap (ABG) (PTA4): 15 dB ± SD 8 dB (EES-); 16 dB ± SD 8 dB (EES+); postoperative ABG (PTA4): 11.25dB ± SD 11.3dB (EES-); 14 dB ± SD 10 dB (EES+)) did not differ significantly (*p* > 0.05) between the two groups. Postoperative hearing results and quality of life tended to improve in both groups (*p* > 0.05).

**Discussion:**

The passive endoscope holder has been successfully applied during the course of the study. However, modifications of the endoscope holder and further studies are recommended focusing on positioning of grafts and prostheses to obtain conclusive results regarding the superiority of two-handed EES over one-handed conventional EES.

**Supplementary Information:**

The online version contains supplementary material available at 10.1007/s00405-024-09018-9.

## Introduction

Endoscopic ear surgery (EES) has grown as a complementary technique to microscopic ear surgery (MES) over the last decades since it was first described in the literature in the 1990s [1–3]. This is reflected in the increasing number of cases in patient care and the growing number of medical device manufacturers developing solutions for EES (especially optics and instrumentation). The growing scientific importance of EES is reflected in a sharp increase in the annual number of scientific publications [4, 5], especially in the last decade, and the establishment of international working groups (e.g. The International Working Group on Endoscopic Ear Surgery, IWGEES) or the organization of medical congresses dedicated exclusively to EES.

Advantages of EES include **improved visualization** [6] of anatomy (e.g. superior and inferior retrotympanon, protympanon, ventilation routes of the tympanic cavity including tensor fold and tympanic isthmus) and pathology in the surgical field by bridging the external auditory canal, placing the lens lateral to the tympanic membrane or within the tympanic cavity, providing a wider angle of view, an improved depth of field and a higher magnification compared to MES. All surgeons and assistants follow the same live image on monitors with the same field of view. This improves the involvement of all participants in the operating room (OR), its **traceability** and has a positive impact on **intraoperative surgical teaching** [7]. Further benefits include **reduced surgical trauma** due to the elimination of external ear drilling or incisions often required with MES, which might result in a reduction in post-operative pain [8–10], and reduced length of stay in the hospital. Children in particular benefit from the use of EES over MES because their short and elongated external ear canals are ideal for endoscope placement [11]. Cut-suture times, postoperative hearing results and complication rates tend to be comparable for both techniques [12–15]. The disadvantages of EES compared to MES are the loss of binocular vision and thus depth perception, which can only be compensated by relative movements of the endoscope in the situs (optical parallax), and the one-handed surgical technique.

To compensate for the latter disadvantage and to address this medical usecase, **active** [16–18] and **passive endoscope holders** [19–29] have been developed and investigated over the last decade.

The aim of this study is to quantify for the first time the benefit of using a novel passive endoscope holder in two-handed EES based on intraoperative process parameters (cut-suture time and operating time) and postoperative outcome parameters (postoperative hearing outcome, graft removal rate, patient-reported outcome measures, postoperative complications) compared to conventional one-handed EES.

## Materials and methods

The single center-study was conducted from December 1, 2021 to June 1, 2024 in a tertiary referral hospital (Department of Otorhinolaryngology, Head and Neck surgery at the University Hospital Carl Gustav Carus at the Technische Universität Dresden) in accordance with the Declaration of Helsinki and according to the rules of good clinical practice, following the review and approval of the local ethics committee at the Technische Universität Dresden (BO-EK-4012022).

### Inclusion criteria

Patients with unilateral tympanic membrane perforation due to history of trauma or chronic otitis media without cholesteatoma (COMsC) who have never had ear surgery before and were considered eligible for endoscopic tympanoplasty (primary procedure) were included after being asked about their willingness to participate in the study and receiving detailed information.

### Exclusion criteria

Patients with chronic otitis media with cholesteatoma (COMwC) and/or patients with a history of ear surgeries and/or with an outer ear canal diameter of less than 6 mm and/or patients who were unable to give informed consent and/or who were unable to follow the study instructions and/or who suffered from neurological/psychological disorders were excluded from participation in the study.

The operations were planned as exclusively endoscopic (EES Class 3 [30]) procedures (tympanoplasty type 1). In case a defect of the ossicular chain (OC) was detected intraoperatively, which required a reconstruction of the OC by means of a partial or total prosthesis, or in case a system change (endoscope to microscope) was required intraoperatively, the data of these patients were excluded postoperatively from the evaluation within the scope of the study.

### Study arms and randomization

Two study arms were established. In the first study arm (EES-), endoscopic tympanoplasty was performed conventionally one-handed. In the second study arm (EES+), tympanoplasty was performed two-handed using the Endofix exo passive endoscope holder (Co. Aktor med GmbH, Neutraubling, Germany), in which the endoscope could be mounted. In both study arms, 0° and 30° otoscopes with a length of 11 cm and a diameter of 3 mm (Co. Spiggle & Theis Medizintechnik GmbH, Overath, Germany) and a full HD camera system (Co. Karl Storz, Tuttlingen, Germany) were used.

Patients who had given their informed consent for participation in the study were alternately assigned to the study arms in a quasirandomized fashion.

### Endofix Exo

The Endofix exo (Co. AKTOR med GmbH, Neutraubling, Germany, https://aktormed.info/en/products/endofix-exo) is a CE-certified passive endoscope support arm for minimally invasive surgery, which can be mounted to the operating table. It is positioned using a magnetic brake system that is released and locked by pressing a button on a control unit located at the distal end near the optic holder and easily accessible to the surgeon.

### Course of surgery und involved surgeons

The endoscopic operations were performed according to the recommendations in the literature [11, 31, 32]. 21 of 25 procedures (EES- study arm, 84%) and 12 of 15 procedures (EES + study arm, 80%) were performed by 3 surgeons (resident: 8 (EES-) and 9 (EES+) procedures; experienced surgeon 1: 6 (EES-) and 2 (EES+) procedures; experienced surgeon 2: 8 (EES-) and 1 (EES+) procedures). 3 procedures each (EES- and EES+) were performed by other experienced surgeons.

### Timing of data collection

The preoperative measurements (audiometric measurements and evaluation of HRQOL) were conducted up to 2 weeks prior surgery. The perioperative data collection was performed during surgery (recording of cut-suture time, operating time) or immediately after its completion (self-designed questionnaire on the surgeon’s satisfaction with the SAS). The postoperative measurements were conducted one day (postoperative audiometric control of BC stability) and 6 months postoperatively (evaluation of PTA, HRQOL, graft take rate, postoperative complications and complication rate).

### Measurements

#### Audiometric measurements

The audiometric measurements were conducted in a soundproof room (DIN EN ISO 8253).

Bone conduction (BC), air conduction (AC) and air-bone gap (ABG) across the frequencies 0.5, 1, 2 and 4 kHz were measured by means of pure tone audiometry and for the ear planned for surgery with an AT900 or AT1000 clinical audiometer (Auritec GmbH, Hamburg, Germany). The mean of the pure-tone audiometry (4PTA) was calculated for BC, AC and ABG. Whenever the labels ‘BC’, ‘AC’, ‘ABG’ appear in the figures in this paper, the corresponding data refers to the mean PTA4.

#### Patient reported outcome measures

The Zurich chronic middle ear inventory 21 (ZCMEI 21) [33] was applied to determine the effect of the performed ear surgery on the increase in subjective health related quality of life (HRQOL) in the context of the present chronic middle ear disease.

#### Recording of operating times

Cut-suture time and total operating time (total duration during which actions were undertaken on the patient or operating table for the realization of the surgery) were recorded.

#### Self-designed questionnaire on surgeon’s satisfaction with the passive endoscope holder

A two-part questionnaire was designed to measure the surgeon’s satisfaction with the passive endoscope holder and the endoscopic surgical technique in general in various dimensions with a total of 9 questions (handling of the system (2 questions), optical display (5 questions) and ergonomics (1 question)) using a 6-point Likert scale in the first part of the questionnaire. In the second part of the questionnaire, the systems are compared with each other by means of the items ‘handling’, ‘visual presentation’ and ‘ergonomics’ using a bilateral scale ranging between − 5 and + 5, with the middle (0) indicating equivalence between the systems.

Please find an English translation of the questionnaire attached in the appendix.

#### Graft take rate and postoperative complications

The graft take rate and postoperative complications: persisting postoperative bone conduction deterioration (defined as a decrease in 3 frequencies > 15dB or 2 frequencies > 20dB), infections, facial nerve palsy, graft failure were evaluated after 6 months.

### Calculations and statistical analyses

All calculations and statistical analyses were performed by means of Microsoft Excel (Microsoft.

Corporation, Redmont, U.S.), Origin (OriginLab, Northampton, U.S.) and SPSS (IBM, Armonk, U.S.). All statistical tests were performed at the significance level of α = 0.05. When the text refers to a trend or tendency, the results are not significant (*p* ≥ 0.05).

The majority of the data are displayed by means of boxplots with the following tendency and dispersion measures: mean, median, 25% (Q1) to 75% (Q3) percentile, lower whisker (Q1 -1.5x interquartile range (IQR)), upper whisker (Q3 + 1.5x IQR).

All post-hoc analysis of the effect sizes were conducted using Hedge’s g*, which was estimated from Hedge’s g in analogy to Eq. 1. The effect sizes were interpreted according to the rules of Cohen (1988) (g*= 0.2, small effect; g* = 0.5, medium effect; g*=0.8, large effect).

Formula for estimating Hedge’s g*:

1 $$\:g*\:\approx\:\:g\:\times\:(1-\frac{3}{4\left({n}_{1}+\:{n}_{2}\right)-9}$$

with

g∗ Hedge’s g*.

g Hedge’s g.

n1 and n2 group sizes (in case of testing only one sample, n2 is excluded from the equation).

#### Audiometric measurements and HRQOL (ZCMEI 21)

Two sided t-test for independent samples was used for the preoperative and postoperative comparison of the mean values between the three groups (BC, AC, ABG, ZCMEI 21 scores) of the two studyarms (EES-, EES+). Previously, the distribution of the individual values (preoperative and postopoperative BC, AC, ABG as PTA4 and ZCMEI 21 scores) between the groups (EES- and EES + respectively) was tested for equality of variance using Levene’s test. In case of a lack of variance homogeneity, the Welch test was applied.

Two sided t-test for paired samples was used for the preoperative and postoperative comparison of the mean values within the audiometric (BC, AC, ABG) and HRQOL (ZCMEI 21) data of the two studyarms (EES-, EES+).

In addition, the differences between pre- and postoperative hearing results in the three groups (BC, AC, ABG) and ZCMEI 21 scores of the two study arms (EES-, EES+) were calculated.

#### Self-designed questionnaire on surgeon’s satisfaction with the passive endoscope holder

Two sided t-test for independent samples was used for the comparison of the mean values of the 9 questions of the first part and the 5 questions of the second part of self-designed questionnaire (questionnaire attached in the appendix).

#### Complications and graft take rate

Chi-squared test (χ2 test) was applied to compare the results of both study arms.

## Results

### Demographic data, etiology of TM perforations

40 patients (EES-: 25; EES+: 15) were enrolled in the study. A statistically significant difference was observed in the mean age of the patients in the study arms (EES-: 28 years; EES+: 48 years) (*p* < 0.05, t-test). The TM perforations were caused either by chronic otitis media without cholesteatoma (COMsC) (EES-: 21/25 cases; EES+: 14/15 cases) or by trauma (EES-: 4/25 cases; EES+: 1/15 cases). Please refer to Table 1 for further details. The complication rate and the graft take rate reported in the table are discussed below.

**Table 1:** Demographic data, etiology of TM perforation and brief overview of postoperative complications and graft take rate

Item	EES-	EES+
Number of patients	25	15
Age (mean ± standard deviation) in years	28.1 ± 21.2	47.7 ± 20.8
Etiology of perforation	- COMsC (21 cases; ear issues since childhood: 12 cases; no exacerbation remembered, dry perforation at initial diagnosis: 3 cases; recurrent exacerbation in adulthood: 6 cases) - Trauma (4 cases)	- COMsC (14 cases; ear issues since childhood: 3 cases; no exacerbation remembered, dry perforation at initial diagnosis: 5 cases; recurrent exacerbation in adulthood: 6 cases) - Trauma (1 case)
Postoperative complications (monitoring: 6 weeks postoperative)	6 of 25 patients (24%)	5 of 15 patients (33%)
Graft take rate (6 month postoperative)	88% (3 recurrent perforations)	80% (3 recurrent perforations, one patient with conditions subsequent to adjuvant radiation)

### Defect sizes, localizations and grafts

**Fig. 1:**
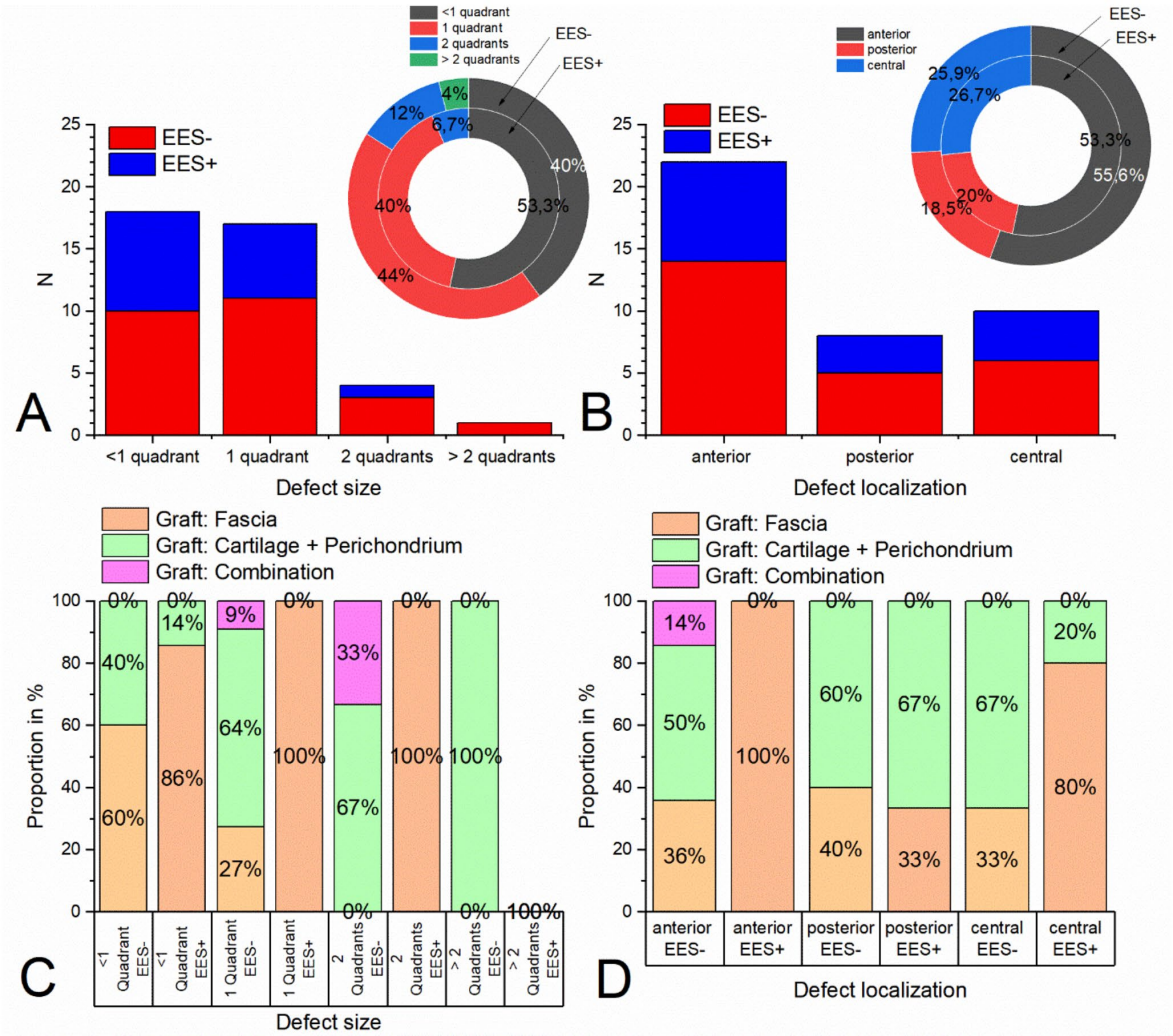
**A** Absolute and relative distribution of TM defect sizes. TM defects of smaller size (< 1 or 1 quadrant) are predominant in both study arms (EES- and EES+). **B** Absolute and relative distribution of TM defect localizations. In both study arms, anterior perforations predominate over posterior and central perforations. **C** TM defect size and applied reconstruction material used. In the EES- arm, the proportion of cartilage and perichondrium or combination grafts increases with the defect size compared to the use of fascia alone; in the EES + arm, fascia is used almost exclusively. **D** TM defect localization and reconstruction material used. In the EES- arm, the use of cartilage and perichondrium dominates in all defect localizations. In the EES + arm, cartilage and perichondrium are increasingly used for posterior perforations, while fascia dominates in all other perforation localizations

In both study arms, mainly small perforations with a maximum of one quadrant (EES-: 84.0%, EES+: 93.3%) were included (please refer to Fig. 1A). The perforations were predominantly located either anteriorly (EES-: 55.6% / EES+: 53.3%) or centrally (EES-: 25.9% / EES+: 26.7%) (please refer to Fig. 1B). While in the EES- study arm the proportion of fascia used as a graft decreased with increasing perforation size and that of cartilage + perichondrium increased, in the EES + study arm fascia was used almost exclusively regardless of the perforation size (please refer to Fig. 1C). In the EES- study arm, regardless of defect location (posterior, central, anterior), approximately 33 to 40 % fascia and approximately 50 to 66% cartilage + perichondrium were used as grafts. In the EES + study arm, fascia was used 100% for anterior perforations, 80% for central perforations (20% cartilage + perichondrium) and 33% for posterior perforations (67% cartilage + perichondrium) (please refer to Fig. 1D).

### Operating times and learning curves

**Fig. 2:**
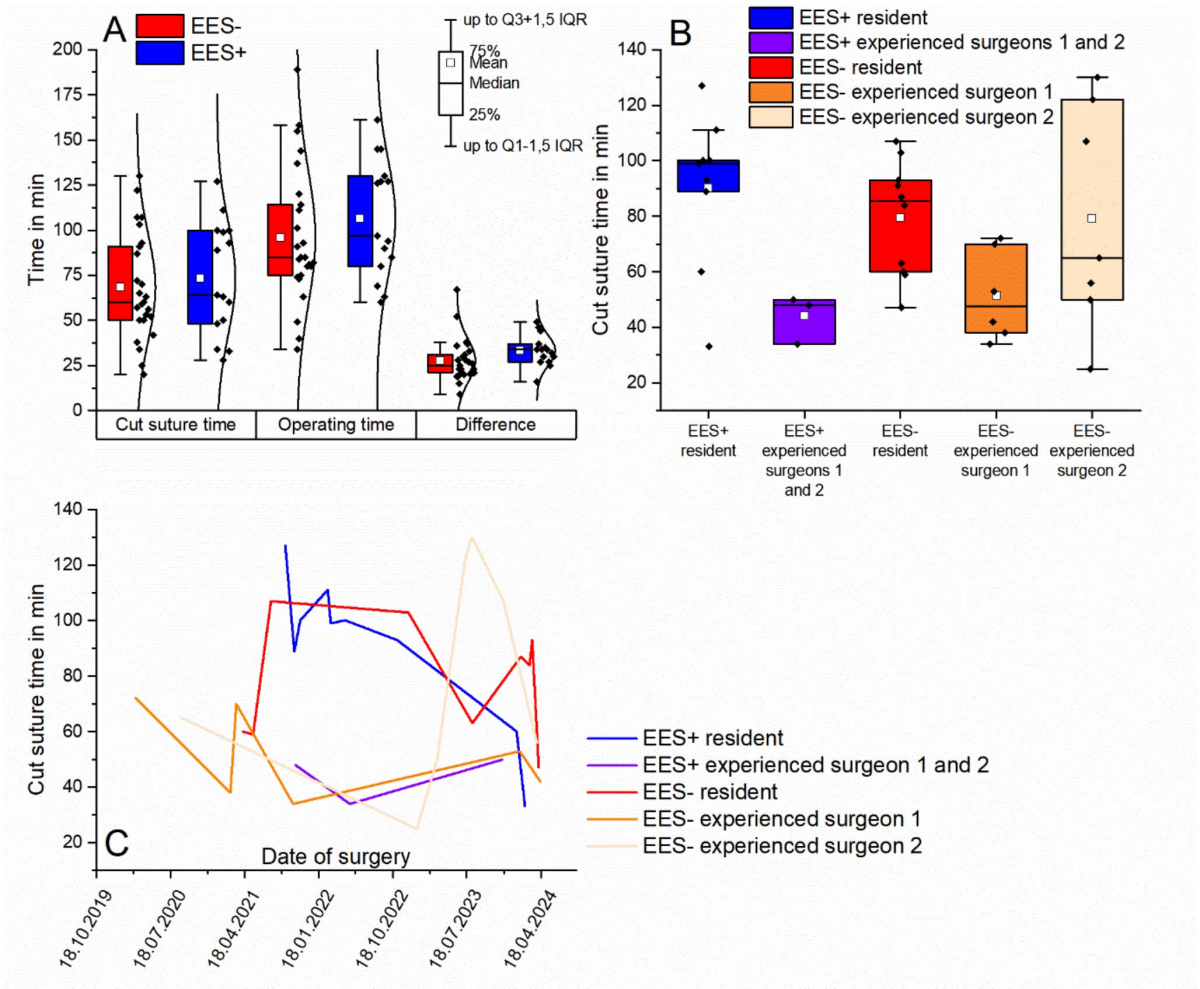
**A** Cut suture time, operating time and difference (operating times – cut-suture times) displayed for both study arms (EES- and EES+). There are no significant differences between the groups. **B** Subgroup analysis of cut-suture times according to surgeon experience (see explanation regarding the EES + subgroup in the continuous text). **C** Learning curves: cut-suture times as a function of surgical expertise over time. The EES- and EES + resident subgroups tended to show a clearer decrease in cut-suture times over the course of the study than the subgroups of experienced surgeons

Both the mean cut-suture times (CST) and the operating times (OT) and the differences (D) between both groups were tendentially shorter in the EES- group (CST: 68.24 ± 29.59 (SD) min, OT: 95.92 ± 37.75 (SD) min, D: 27.68 ± 11.96 (SD) min) than in the EES + group (CST: 73.26 ± 31.39 (SD) min, OT: 106.60 ± 32.57 (SD) min, D: 33.33 ± 8.50 (SD) min) (please refer to Fig. 2A). In both the EES- and EES + study arms, the mean cut-suture times of the resident (EES-: 82.88 ± 19.50 (SD) min; EES+: 90.22 ± 27.95 (SD) min) was higher than that of the two experienced surgeons (EES- experienced surgeon 1: 51.50 ± 16.93 (SD) min; EES- experienced surgeon 2: 79.29 ± 40.24 (SD) min; EES + experienced surgeons: 44.00 ± 8.72 (SD) min) (please refer to Fig. 2B). However, the resident’s cut-suture times tended to show a learning curve, while the times of the experienced surgeons were already rather low during their first procedures (please refer to Fig. 2C).

### Surgeon’s satisfaction with the passive endoscope holder

**Fig. 3:**
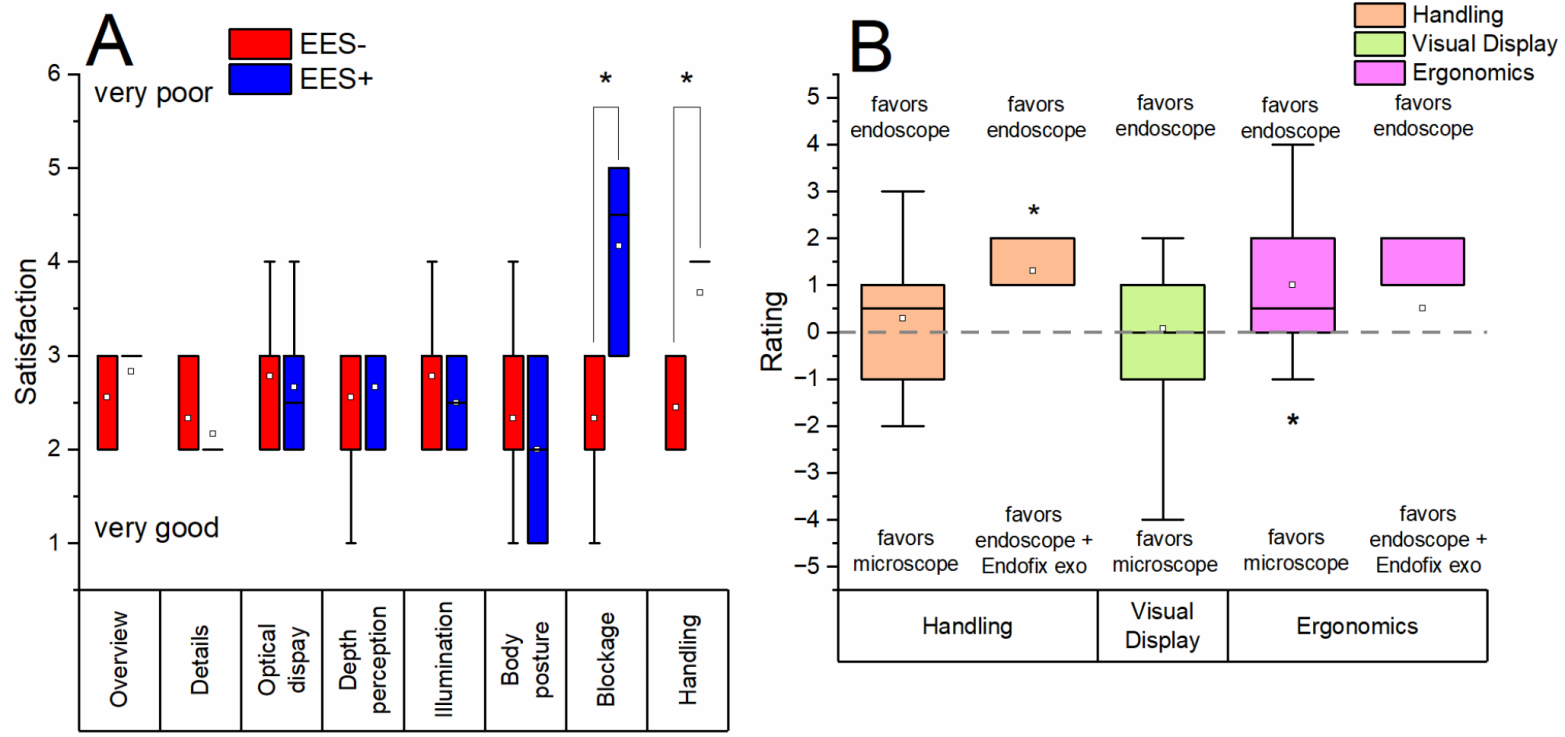
**A** Postoperative survey on surgeon satisfaction with the applied system (EES- or EES+). In the EES + group, satisfaction regarding the items ‘blockage’ and ‘handling’ was statistically significantly worse than in the EES- group. Asterisc (*) indicates statistical significant differences between the study arms. **B** Comparison of differenct visualisation systems (microscope vs. endoscope (EES-) vs. endoscope + Endofix exo (EES+)) regarding the items ‘handling’, ‘visual display’ and ‘ergonomics’. Statistically significant advantage in favour of EES- over EES + regarding the item ‘handling’ and in favour of microscope over endoscope regarding the item ‘ergonomics’. Asterisc (*) indicates statistical significant difference from reference line

There were no statistical significant differences between the study arms for the items ‘overview’, ‘details’, ‘optical display’, ‘depth perception’, ‘illumination’ and ‘body posture’ (all items ranked on average between grade 2 and 3), although the item ‘body posture’ tended to be rated slightly better on average in the EES + arm than in the EES- arm (please refer to Fig. 3A) (p ≥ 0.05, independent samples t-test). However, the EES + study arm was rated statistically significantly inferior on average compared to the EES- study arm for the items ‘blockage’ (4.2 ± 1.0 (SD) / 2.3 ± 0.7 (SD), p = 0.001; t-test for independent samples; g* = 0.82, large effect) and ‘handling’ (3.7 ± 0.8 (SD) / 2.4 ± 0.5 (SD), *p* = 0.012; t-test for independent samples; g* = 0.65, large effect).

The questionnaire was also used to compare the handling of the endoscopic procedures (EES+/EES-) with the microscopic surgical technique that is otherwise routinely performed by the surgeons (outside of this study).

With regard to the overall comparison of the systems (please refer to Fig. 3B), there is a tendential mean advantage in handling for the endoscope alone compared to the microscope (0.29 ± 1.38 (SD), *p* ≥ 0.05; t-test for one sample) and a statistically significant mean advantage compared to the combined use of endoscope + Endofix exo (1.3 ± 1.25 (SD), *p* = 0.009; t-test for one sample; g* = 1.24, large effect). There is no difference in average visual display between the endoscope and the microscope (0.07 ± 1.64 (SD), *p* ≥ 0.05; t-test for one sample). There is a statistically significant mean difference in ergonomics in favour of the endoscope alone (1.00 ± 1.96 (SD), *p* = 0.016; t-test for one sampe; g* = 1.35, large effect) compared to the microscope and a average trend in favour of the endoscope alone compared to the combined use of endoscope + Endofix exo (0.50 ± 1.96 (SD), *p* ≥ 0.05; t-test for one sample).

### Audiometric results

**Fig. 4:**
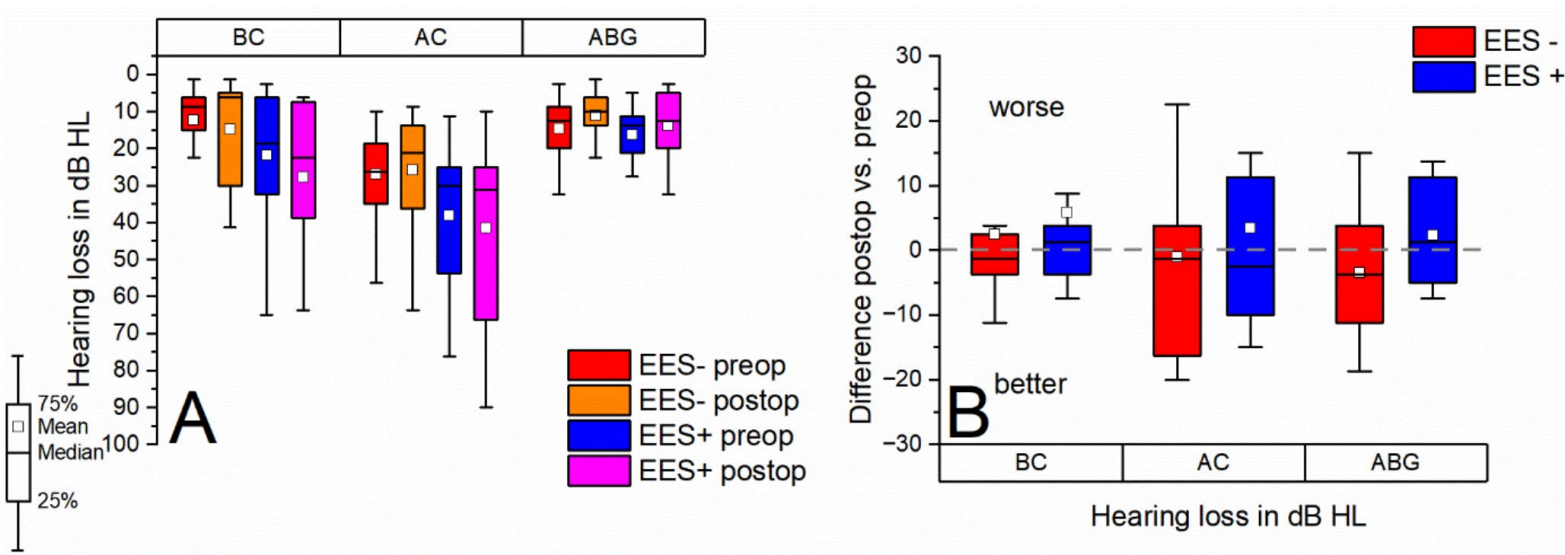
**A** Pre- and postoperative hearing loss (AC, BC and ABG each as PTA4) for both study arms (EES- and EES+). No statistical significant differences between the ams across all items. Tendentially higher mean preoperative and postoperative hearing loss (BC and AC) in the EES + group. **B** Difference between post- and preoperative hearing loss (AC, BC and ABG each as PTA4) for both study arms. No statistical differences between the two arms, mean results tended to be superior in the EES- arm

**Preoperatively**, the mean BC/AC/ABG (please refer to Fig. 4A) tended to be worse in the EES + group (21.93 ± 19.86 dB (SD) / 38.18 ± 21.78 dB (SD) / 16.25 ± 7.58 dB (SD)) than in the EES- group (12.35 ± 10.08 dB (SD) / 26.91 ± 12.03 dB (SD) / 14.56 ± 7.68 dB (SD)) (*p* ≥ 0.05, t-test for independent samples). **Postoperatively**, mean BC/AC/ABG remained tendentially worse in the EES + study arm (27.72 ± 20.23 dB (SD) / 41.59 ± 26.05 dB (SD) / 13.86 ± 9.58 dB (SD) than in the EES- study arm (14.85 ± 14.70 dB (SD) / 25.88 ± 16.20 dB (SD) / 11.02 ± 7.43 (SD)) (*p* ≥ 0.05, t-test for independent samples). The mean BC/AC/ABG tended to deteriorate in the EES + study arm postoperatively compared to preoperatively (difference: 5.7 ± 19.06 dB (SD) / 3.41 ± 21.07 dB (SD) / 2.39 ± 8.07 dB (SD); *p* ≥ 0.05, one sample t-test and *p* ≥ 0.05, t-test for dependent samples - please refer to Fig. 4B). The EES- study arm tended to show an improvement in mean postoperative AC (-1.03 ± 16.25 dB (SD)) and ABG (-3.53 ± 9.50 dB (SD)) with a concomitant tendential deterioration in mean postoperative BC (2.5 ± 11.36 dB (SD)) (*p* ≥ 0.05, one sample t-test and *p* ≥ 0.05, t-test for dependent samples). The BC/AC/ABG differences were not statistically significantly different between the two study arms (*p* ≥ 0.05, t-test for independent samples).

### Patient reported outcome measures – ZCMEI 21

**Fig. 5:**
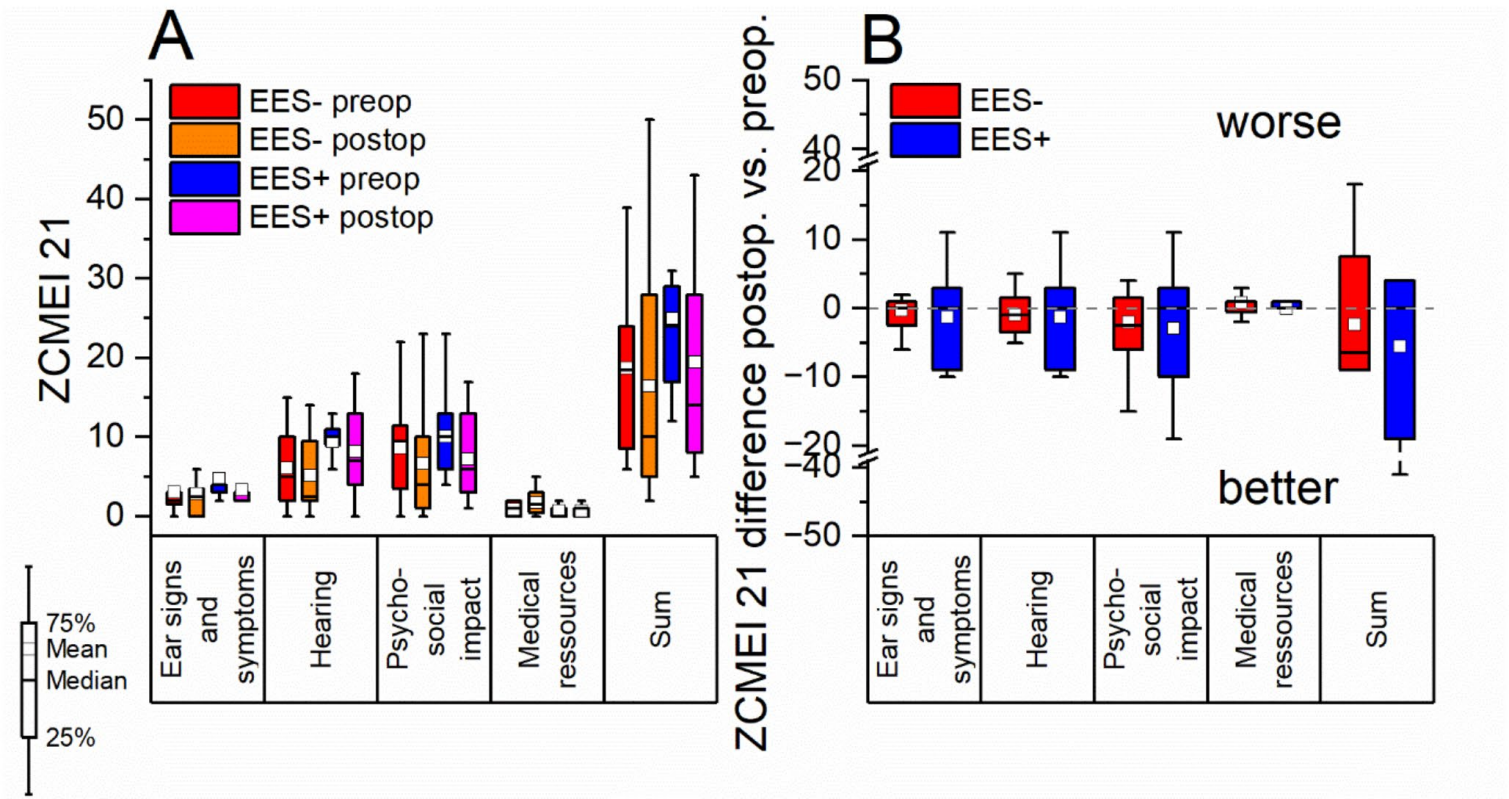
**A** Pre- and postoperative HRQOL (ZCMEI 21) for both study arms (EES- and EES+). Higher scores indicated a poorer HRQOL. No statistical significant mean differences between both arms across all items. Tendentially worse preoperative mean HRQOL in the EES + group. **B** Difference between post- and preoperative HRQOL. No statistical significant differences between both arms across all items. Tendentially superior average HRQOL in the EES + arm except for the item ‘medical ressources’

**Preoperatively**, the mean overall score (sum) in the ZCMEI-21 (please refer to Fig. 5A) tended to be worse in the EES + group (25.00 ± 10.70 (SD)) than in the EES- group (18.75 ± 11.01 (SD)) (*p* ≥ 0.05, independent samples t-test). This was also confirmed for all specific dimensions (ear signs and symptoms; hearing; psychosocial impact; medical resources (all *p* ≥ 0.05, independent samples t-test). **Postoperatively**, the mean overall score (sum) tended to improve in the EES + group (19.44 ± 14.48 (SD)) and in almost all specific dimensions (exception: ‘medical ressources’) compared to preoperatively (*p* ≥ 0.05, t-test for dependent samples), but was still inferior to the EES- group (16.41 ± 15.76 (SD)) (*p* ≥ 0.05, independent samples t-test). The EES- group also tended to show an improvement in the mean overall HRQOL (sum) and in almost all (exception: ‘medical ressources’) the specific dimensions (*p* ≥ 0.05, t-test for dependent samples).

In the EES + group, the mean **postoperative improvement** of the ZCMEI-21 (please refer to Fig. 5B) was tendentially higher than in the EES- group, both in the total score (postoperative difference EES+: -5.56 ± 21.21 (SD), postoperative difference EES-: -2.33 ± 11.43 (SD); *p* ≥ 0.05, independent samples t-test) and in all specific dimensions. The mean improvement in the total score in the EES study arm thereby exceeded the **minimum clinically important difference of the ZCMEI 21** (5 points) [33]. However, in neither of the two study arms the average improvements in the total score or the specific dimensions were statistically significantly different from the reference line (y = 0; *p* ≥ 0.05, one sample t-test).

### Postoperative complications and graft take rate

The overall **complication rate** tended to be higher in the EES + arm (5 of 15 patients, 33%) than in the EES- arm (6 of 25 patients, 24%) (χ2 test, *p* > 0.05). In the EES- group, two *peripheral facial nerve pareses* occurred, one early (< 24 h postoperatively, patient 4) and one late (patient 6). Both pareses were associated with postoperative BC deterioration. During revision surgery of the early paresis, a hematoma was drained that was ballooning the tympanic cavity and compressing the tympanic segment of the facial nerve. The late paresis occurred in the context of a postoperative ear infection, and recurrent perforation was noted during follow-up.

Two further *BC deteriorations* occured in both study arms (EES-: patients 5 and 9; EES+: patients 10 and 24), patient 24 (EES-) was treated for postoperative infection, all other BC deteriorations remained unsolved. There were three additional *recurrent TM perforations* in the EES- group (one documented postoperative infection) and two in the EES + group.

All infections were treated with intravenous or oral antibiotics; in the case of BC deterioration, i.v. corticosteroid therapy (250 mg Prednisolone once a day for 3–5 days) was administered.

The **graft take rate** tended to be lower in the EES + arm (12 of 15 cases, 80%) than in the EES- arm (22 of 25 cases, 88%) (χ2 test, *p* > 0.05). Please refer to Tables 1 and 2 for detailed information regarding postoperative complications and graft take rate.

**Table 2:** Overview of all patients with postoperative complications and graft failure, summary of the complications, the graft applied and the surgeon’s experience

Item Patient	Age in years	Postoperative BC deterioration (initial deterioration in dB (PTA4) / complete remission)	Postoperative infection	Postoperative peripheral facial nerve paresis	Recurrent perforation	Graft (fascia (1), cartilage + perichondrium (2))	Surgeon (resident (1), experienced surgeon 1 (2), experienced surgeon 2 (3), other (4))
		Monitoring: 6 weeks postoperatively	Monitoring: 6 month postoperatively				
1 (EES+)	46	No	Yes	No	Yes	2	1
5 (EES+)	33	Yes (15dB / yes)	No	No	No	1	1
6 (EES+)	40	No	No	No	Yes	1	1
7 (EES+)	55	No	No	No	Yes	1	1
9 (EES+)	33	Yes (34dB / no)	No	No	No	1	4
1 (EES-)	14	No	No	No	Yes	1	2
4 (EES-)	37	Yes (11dB / no), hematoma	No	Yes (early palsy, complete remission)	No	1	4
6 (EES-)	24	Yes (19dB / no)	Yes	Yes (late palsy, complete remission)	Yes	2	1
10 (EES-)	66	Yes (9dB / no)	No	No	No	1	2
23 (EES-)	5	No	No	No	Yes	2	3
24 (EES-)	65	Yes (23dB / no)	Yes	No	No	1	2

## Discussion

The aim of this study was to quantify the benefit of using a novel passive endoscope holder as an SAS in two-handed EES (Endofix exo, Co. Aktor med GmbH, Neutraubling, Germany) based on intraoperative process parameters and postoperative outcome parameters compared to conventional one-handed EES for the first time. In summary, the study protocol used here was not able to show an advantage for the use of the endoscope holder in the context of two-handed EES compared to conventional one-handed EES. Nevertheless, the authors are convinced that the endoscope holder presented here offers a promising perspective for two-handed EES.

### Postoperative outcome – our data compared to a review of the literature

To the authors’ knowledge, there is no literature data comparing one-handed EES with two-handed EES. However, there are several studies comparing EES with MES in tympanoplasty type 1 [34–37]. Furthermore, a single center has published data from extensive experience with two-handed EES [24, 27, 38, 39].

#### Graft take rate

In the literature, the **GTR** for conventional EES and MES is between 83 and 100%, depending on the study [34–36], size of the perforations, autologous reconstruction material (cartilage and/or fascia) used and reconstruction technique applied. There is no clear advantage to either of the two surgical techniques. In the two-handed EES, the GTR was 95–97%. Tragal cartilage and perichondrium reinforced by temporal fascia was used in the majority of cases and positioned in underlay technique [24, 27, 38, 39].

GTR in our study was 88% and at least satisfactory in the EES- study arm (80% in the EES + study arm) and close to mean data reported in the literature. Temporal fascia was used as reconstruction material in 3 of 5 recurrent perforations (across both study arms). There is a tendency in the literature [40] that the use of cartilage is associated with a lower likelihood of recurrent perforation than the use of fascia.

#### Postoperative hearing results

The **postoperative hearing results** revealed an ABG reduction of 9 to 20 dB with stable BC and no significant differences between MES and EES [34–37]. In two-handed EES, the reduction in ABG was between 21 and 23 dB [24, 27, 38, 39].

The mean postoperative hearing results in our study did not show any significant improvement, at least in the EES + study arm. If the postoperative hearing results in both groups are adjusted for patients with severe postoperative BC deterioration (EES+: 2 patients, EES-: 3 patients), the ABG (PTA4) decreases by approx. 5 dB (EES-: 15 dB to 10 dB; EES+: 16 dB to 11 dB) in both study arms while the BC threshold remains stable. Considering that 84% (EES-) and 93.3% (EES+) of the patients exhibited only a quadrant-sized perforation preoperatively and thus no significant reduction of the AC/ABG component was to be expected during tympanoplasty, the results appears acceptable. The remaining ABG may be attributed to tympanosclerosis processes due to chronic inflammation.

#### Postoperative health related quality of life

There is only one study of acceptable quality comparing **HRQoL** of MES to EES using the ZCMEI-21 which was applied in our study, too [37]. The postoperative ZCMEI total scores of both groups were significantly reduced (MES: 25 to 15, EES: 23 to 10), especially the scores for middle ear symptoms (MES and EES 5 to 2), subjective hearing (MES: 7 to 4, EES: 6 to 3) and psychosocial impact (MES: 9 to 5, EES hearing (MES: 7 to 4, EES: 6 to 3). The scores for medical resource utilization did not change significantly (*p* > 0.05). In comparison to the postoperative outcomes of MES, the total scores and the psychosocial impact scores were significantly lower in EES (*p* < 0.05). There is no data on HRQoL in two-handed EES.

Our data also show a trend towards an improvement in HRQoL in the total score and in individual subscores, but our results do not show any significance. This is possibly favored by a disproportion of small group sizes and a relatively high proportion of postoperative complications, which negatively influence the overall results.

The following sections address the limitations of the current SAS and the study presented here. Suggestions for modifying the system and starting points for further investigations are provided.

### Comparison of the endofix exo with current endoscope holding arms in ENT

Holding arms represent the lowest level of sophistication within the classification of surgical assistance systems designed to guide surgical instruments. In head and neck surgery, they must be distinguished from single-arm (e.g. *Robotol*, Co. Collin medical, France) or multi-arm (e.g. *Da Vinci SP*, Co. Intuitive Surgical, USA) master-slave systems, which are primarily telemanipulated, and semi-automated surgical robots (e.g. *HEARO*, Co. Cascination, Switzerland). Active holding arms can be telemanipulated using an external console (e.g. joystick or spacemouse), while passive holding arms are manipulated using muscle power (possibly supported by auxiliary motors in the holding arm) after a locking mechanism has been released.

Passive endoscope support arms in head and neck surgery range from the simplest developments of rod combinations [19] and custom modifications of commercially available camera mounts [21] to more complex proprietary designs that increase maneuverability e.g. through the use of gas springs and rack and pinion [26, 27]. They culminate in complex industrial solutions with multiple degrees of freedom and sophisticated stabilization and locking mechanism (e.g. pneumatic nitrogen fail-safe brakes system [20]). The Endofix exo belongs to the latter category. After unlocking a magnetic brake system with spring force compensation via a controller magnetically attached to the holding arm, the arm can be swiveled freely, with the number of degrees of freedom increasing from proximal to distal.

### Inferiority of the two-handed EES compared to the one-handed EES: cut-suture times, operating times and subjective satisfaction of surgeons – suggestions for modifications of the system

Due to the sophistication of the Endofix exo as a medical device, the surgeons in the study had high expectations for the functionality of the SAS. However, some shortcomings prevented the SAS from prevailing over conventional EES in terms of cut-suture times, operating times, handling and ergonomics (please refer to Fig. 3). The maneuverability in the external auditory canal and in the tympanic cavity is limited due to the anatomical dimensions. Positioning the endoscope and two surgical instruments was therefore a challenge. In many cases of the EES + studyarm, we experienced collisions between the endoscope and at least one of the instruments, which were also enhanced by the lack of haptic feedback. Usually, the endoscope blocked the range of movement of the instrument in the non-dominant hand. In conventional EES, the collision of instrument and endoscope causes haptic feedback and usually results in involuntary repositioning of the endoscope and/or instrument. However, this does not occur in two-handed EES using an endoscope that is statically positioned in the external auditory canal. During repositioning, an imbalance of the distal end effector that fixated the endoscope became evident. When the locking mechanism was released, the end effector followed the force of gravity and dropped unless it was held in position by muscle force. Repositioning in the external ear canal therefore had to be conducted with extreme care to avoid causing serious injury to the tympanic membrane and middle ear structures with the endoscope. After repositioning the endoscope and locking the arm, an imbalance was observed again, resulting in an unfavorable drop of the end effector by a few millimeters. Changing the endoscope was subjectively time-consuming, as the endoscope had to be locked in the endoscope mount using a screw. Overall, operating in the EES + arm was therefore subjectively less dynamic and subjectively more cumbersome than in the EES- arm. These limitations may have led to an increase in cut-suture times and operation times in the EES + study arm compared to the EES- study arm, particularly for procedures performed by the resident who already had fundamental knowledge in lateral skull base surgery (see Fig. 2A and B).

We suggest integrating an active component and a force sensor into the holding arm. The control could be realized via a joystick, a spacemouse and/or a foot switch. Intelligent software linked to the force sensor could lead to automated repositioning of the endoscope while maintaining the visual axis as soon as sufficient contact of an instrument with the endoscope is detected indicating that its range of motion is blocked. A universal quick-release fastener should also be developed for the endoscope mount.

Furthermore, in order to perform two-handed EES safely with an endoscope of 3 mm, the minimum diameter of the external auditory canal should measure at least 6 mm. This finding is in line with the suggestions of Veleur [18].

### Limitations of the study protocol - recommendations for further studies regarding the application of the holding arm for graft and prosthesis positioning

The study was conducted in a clinic with extensive expertise in lateral skull base surgery but limited experience in endoscopic ear surgery at the beginning of this study. In order to facilitate the transition from two-handed microscopic ear surgery to two-handed and one-handed endoscopic ear surgery, the aforementioned passive endoscope holder was purchased. However, during the course of the study, and particularly in the EES- arm, it was determined that the only surgical step in which a two-handed approach may be superior to a single-handed approach, particularly in tympanoplasty type 1, is the reconstruction of the tympanic membrane and the positioning of the grafts [41]. All other steps of the operation, in particular, the freshening of the perforation edges, the preparation of the tympanomeatal flap, the inspection of the tympanic cavity and the inspection and widening of the ventilation and drainage routes could be easily performed with one hand. The use of an aspirator in the non-dominant hand was compensated for by the use of instruments with integrated suction, and bleeding control was achieved by the use of adrenaline-soaked swabs. Even in tympanoplasties with reconstruction of the ossicular chain (operations performed during the study period, but data excluded from the study), it was subjectively found that the advantage of the 2-handed EES was only given when inserting the prosthesis [42]. For future studies on the benefits of two-handed EES, the authors therefore recommend establishing a study protocol that analyzes the different surgical steps separately and focuses on the insertion of grafts and prostheses.

### Patient-related complications in both arms of the current study

Compared to the literature, the complication rate was high in both study arms (EES-: 6/25 patients, 24% and EES+: 5/15 patients, 33%). Marchioni reported a complication rate of 4.1% in 825 EES procedures [43]. However, the complications in both study arms occurred preferentially during the first 10 operations in each case. Considering that the clinic’s experience with the EES was limited at the beginning of the study, the decreasing rate of complications can be interpreted as a learning curve.

2 patients in the EES + arm suffered from a non-reversible postoperative BC deterioration without further symptoms such as dizziness or tinnitus, without a specific etiology (e.g. postoperative infection or hematoma in the tympanic cavity) being found. In the literature [44], a correlation between heat radiation in EES (duration/intensity) and postoperative BC deterioration is propagated. Since both cases occurred in the EES + arm, it is reasonable to speculate that although the tympanic cavity was irrigated regularly to reduce the temperature in the tympanic cavity [32], the inner ear may have suffered thermal damage due to the permanent positioning of the light source in the external ear canal.

### Costs and targeted cost-benefit ratio of applying the Endofix Exo in endoscopic ear surgery

The endoscope holding arm mentioned above was purchased at a price ranging from 40.000 to 50.000 € including accessories and sales tax. The cost-benefit ratio (CBR, please refer to Eq. 2) is a method for calculating the long-term benefit of an investment.

Cost-benefit-ratio

2 $$\:Cost{\text{-}}Benefit{\text{-}}Ratio\:\left(CBR\right)=\:\frac{Total\:Benefit}{Total\:Costs}$$

The total benefit is categorized into direct benefits (e.g., savings from reduced operating time, reduced error rate and costs for complications and follow-up surgeries, reduced length of patient hospital stay) and indirect benefits (increased patient satisfaction, improved postoperative outcome, improved hospital reputation, increased efficiency, savings from fewer consumables with fewer complications). The **reduction in operating time** is the only cost-saving factor that could be calculated in the study. In the clinic conducting the study, the staff and material **costs per minute of surgery**, according to internal billing (ENT physician services, ENT surgical nursing, material costs), were approximately **€7.14**. The costs of anesthesia are not included here. Unfortunately, the study could not (yet) show a reduction in operating time. With extended use of the endoscope in the context of further surgeries (e.g. COMsC / COMcC surgery with OC reconstruction), a reduction in operating time of about 15 min would be desirable. At least 100 operations should be performed per year.

The total costs consist of the acquisition (initial) costs and the operating costs. The initial costs include: purchase price, installation and training costs, technical adjustments or additional infrastructure. The endoscope holder is to be depreciated over 10 years. The initial annual costs thus amount to 4.000–5.000€. The operating costs include: costs for consumables per operation, maintenance costs, software updates or license fees, personnel and training costs, energy consumption. The only significant operating costs per surgery is for the disposable sterile drapes (**16**,**50 € / drape including sales tax**). All other costs are non-existent or negligible.

The **targeted total benefit** is approximately **11.000€/year** (100 surgeries x (15-minute time saving x 7.14€/surgical minute)). The **targeted total costs** are approximately **6000 to 7000 €/year** (1000 surgeries x 16.50 € costs per surgical drape + 4000 to 5000 € / endoscope holder). The targeted CBR is between 1.6 and 1.8. The authors are aware of the limitations of this calculation due to multiple influencing factors, so the calculated CBR is used only as an internal target.

## Conclusion

As part of a study on the performance of two-handed EES compared to conventional one-handed EES in type I tympanoplasty, the innovative passive endoscope holder “Endofix exo” was successfully applied. The study did not show any significant advantages for the application of the passive endoscope holder in terms of cut-suture times, operating times or surgeon’s satisfaction. In addition, some shortcomings of the SAS and the surgical technique have been revealed. Nevertheless, we are convinced of the method of two-handed EES and believe that further studies with different focus (e.g. positioning of prostheses and positioning of grafts in the context of reconstruction of the tympanic membrane) and a modification of the SAS are needed to obtain conclusive results regarding the advantage of two-handed EES compared to one-handed conventional EES.

## Electronic supplementary material

Below is the link to the electronic supplementary material.

Supplementary Material 1

## Data Availability

The processed raw data of all patients/subjects are available on request.
