# Supplementary material for: Conventional one-handed compared to two-handed endoscopic ear surgery using an endoscope holder: a single center study
Source: Eur Arch Otorhinolaryngol. 2024 Oct 15;282(3):1217–30. doi: 10.1007/s00405-024-09018-9 (PMC11890327; doi:10.1007/s00405-024-09018-9)
Supplement: Supplementary file 1 — Supplementary Material 1 [file 405_2024_9018_MOESM1_ESM.docx]

# Questionnaire for surgeons – ear surgery – SAS ENDOFIX exo

**ID Patient:in:**

**ID Operateur:in:**   **Date:**

**Type of surgery:** O Tympanoplasty type I O Others (type:________________________)
Application of: O SAS (ENDOFIX exo)  O Endoscope
 O Microscope (Type: _____________________)

|  | 1 | 2 | 3 | 4 | 5 | 6 |  | Not applicable |
| --- | --- | --- | --- | --- | --- | --- | --- | --- |

1. **What was your overall satisfaction with the handling of the SAS (ENDOFIX exo)?**

| Very satisfied | O | O | O | O | O | O | Unsatisfied | O |
| --- | --- | --- | --- | --- | --- | --- | --- | --- |

1. **How satisfactory was the overview?**

| Very good | O | O | O | O | O | O | Very bad | O |
| --- | --- | --- | --- | --- | --- | --- | --- | --- |

1. **How satisfactory was the visibility of details (anatomical structures, pathologies etc.)?**

| Very good | O | O | O | O | O | O | Very bad | O |
| --- | --- | --- | --- | --- | --- | --- | --- | --- |

1. **How satisfactory was the optical display (e.g. color accuracy, resolution)?**

| Very good | O | O | O | O | O | O | Very bad | O |
| --- | --- | --- | --- | --- | --- | --- | --- | --- |

1. **How satisfactory was the handling of the system?**

| Very good | O | O | O | O | O | O | Very bad | O |
| --- | --- | --- | --- | --- | --- | --- | --- | --- |

1. **How comfortable have you been with your posture during the operation?**

| Very good | O | O | O | O | O | O | Very bad | O |
| --- | --- | --- | --- | --- | --- | --- | --- | --- |

1. **How often have you been impaired by parts of the optical system?**

| Never | O | O | O | O | O | O | Always | O |
| --- | --- | --- | --- | --- | --- | --- | --- | --- |

1. **How satisfactory were you with the depth display of the 2D image?**

| Very good | O | O | O | O | O | O | Very bad | O |
| --- | --- | --- | --- | --- | --- | --- | --- | --- |

1. **How satisfied were you with the illumination of the field of view?**

| Very good | O | O | O | O | O | O | Very bad | O |
| --- | --- | --- | --- | --- | --- | --- | --- | --- |

1. **Did the surgical technique have to be changed during the operation?**

O **Yes:** O Endoscope + ENDOFIX exo 🡪 Endoscope
 O Endoscope + ENDOFIX exo 🡪 Microscope
 O Endoscope 🡪 Microscope

O **No**

**Final evaluation**

**Which system do you think is better overall in terms of handling?**

Microscope superior

|  |  |  |  |  |  |  |  |  |  |
| --- | --- | --- | --- | --- | --- | --- | --- | --- | --- |
|  |  |  |  | equal |  |  |  |  |  |

Endoscope
superior

|  |  |  |  |  |  |  |  |  |  |
| --- | --- | --- | --- | --- | --- | --- | --- | --- | --- |
|  |  |  |  | equal |  |  |  |  |  |

Endoscope + ENDOFIX exo superior
besser

Endoscope
superior

**Which system do you think is better in terms of visual display?**

|  |  |  |  |  |  |  |  |  |  |
| --- | --- | --- | --- | --- | --- | --- | --- | --- | --- |
|  |  |  |  | equal |  |  |  |  | Endoscope superior |

Microscope superior

**Which system do you think is better in terms of ergonomics?**

|  |  |  |  |  |  |  |  |  |  |
| --- | --- | --- | --- | --- | --- | --- | --- | --- | --- |
| Microscope superior |  |  |  |  |  |  |  |  | Endoscope superior |

equal

|  |  |  |  |  |  |  |  |  |  |
| --- | --- | --- | --- | --- | --- | --- | --- | --- | --- |
|  |  |  |  | equal |  |  |  |  |  |

Endoscope + ENDOFIX exo superior
besser

Endoscope
superior

**General questions about the surgeon's level of training**

**I have experience with the following systems:**

**O Microscope**

O > 100 procedures

O < 100 procedures

**O Endoscope**

O > 10 procedures

O < 10 procedures

**O Endoscope + ENDOFIX exo**

O > 10 procedures

O < 10 procedures
